# Predictive value for weakness and 1-year mortality of screening electrophysiology tests in the ICU

**DOI:** 10.1186/cc14554

**Published:** 2015-03-16

**Authors:** H Van Mechelen, G Hermans, F Bruyninckx, T Vanhullebusch, B Clerckx, P Meersseman, Y Debaveye, MP Casaer, A Wilmer, PJ Wouters, I Vanhorebeek, R Gosselink, G Van den Berghe

**Affiliations:** 1 KU Leuven, Belgium; 2 UZ Leuven, Belgium

## Introduction

Muscle weakness in long-stay ICU patients contributes to 1-year mortality [1]. Whether electrophysiological screening is an alternative diagnostic tool also in unconscious/uncooperative patients remains unknown. The aims of this study were to determine the diagnostic properties of abnormal compound muscle action potential (CMAP), sensory nerve action potential (SNAP) and spontaneous electrical activity (SEA) for Medical Research Council (MRC)-sum score defined weakness and their predictive value for 1-year mortality.

## Methods

Data were prospectively collected during the EPaNIC trial (http://ClinicalTrials.gov NCT00512122) [2] from October 2008 to November 2010. From day 8 onwards, nerve conduction studies and electromyography were performed weekly in 642 long-stay and 88 randomly selected short-stay patients and muscle strength was assessed in cooperative patients using the MRC-sum score. The electrophysiologist was blinded for the clinical assessments of the physiotherapists and *vice versa*. The two primary outcomes were: sensitivity, specificity, positive and negative predictive values of abnormal CMAP, SNAP and SEA for weakness (MRC-sum score <48); and the predictive value for 1-year mortality of abnormal findings on first electrophysiological screening. This association was assessed by univariate and multivariate analyses correcting for weakness and other risk factors, including baseline risk factors, comorbidities, illness severity and ICU exposures.

## Results

A total of 730 patients were electrophysiologically screened, of which 432 were tested for weakness. On day 8, only normal CMAP excluded weakness with a high negative predictive value (80.5%). By day 15, abnormal SNAP and the presence of SEA revealed a high positive predictive value (91.7% and 80.0%, respectively). On day 8, only a reduced CMAP was associated with higher 1-year mortality (35.6% vs. 15.2%, *P *< 0.001). This association remained significant after correction for weakness and other risk factors (OR: 2.463 (95% CI: 1.113 to 5.452), *P *= 0.026). Also among conscious/cooperative patients without weakness, reduced CMAP was independently associated with a higher likelihood of death within 1 year (HR: 2.818 (95% CI: 1.074 to 7.391), *P *= 0.035).

## Conclusion

The diagnostic properties of electrophysiological screening vary over time. Abnormal CMAP documented early during critical illness carries information about longer-term outcome, which should be further investigated mechanistically.

## Acknowledgement

HVM and GH contributed equally.
